# Cu^1+^, but not Cu^2+^ is capable of inhibition of AQP4 permeability in an *in vitro* CHO cell based model

**DOI:** 10.1016/j.bbrep.2021.101132

**Published:** 2021-09-14

**Authors:** Arno Vandebroek, Masato Yasui

**Affiliations:** Keio University School of Medicine, Department of Pharmacology, 35 Shinanomachi, Shinjuku, Tokyo, 160-8582, Japan

**Keywords:** Aquaporin 4, Inhibitor, Proteoliposome, Osmotic water permeability, Cu(I)Cl

## Abstract

Aquaporin 4 (AQP4) is an important water channel in the central nervous system which is implicated in several neurological disorders. Due to its significance, the identification of molecules which are able to modulate its activity is quite important for potential therapeutic applications. Here we used a novel screening method involving CHO cell lines which stably express AQP4 to test for potential molecules of interest. Using this method we identified a metal ion, Cu^1+^, which is able to inhibit AQP4 activity in a cell model, an interaction which has not been previously described. This inhibition was effective at concentrations greater than 500 nM in the CHO cell model, and was confirmed in a proteoliposome based model. Furthermore, the binding sites for Cu^1+^ inhibition of AQP4 are identified as cysteine 178 and cysteine 253 on the intracellular domain of the protein via the synthesis of AQP4 containing point mutations to remove these cysteines. These results suggest that Cu^1+^ is able to access intracellular binding sites and inhibit AQP4 in a cell based model.

## Highlights

• Cu^1+^ inhibits water permeability through AQP4 in a novel *in vitro* cell model.
• Inhibition is mediated by binding to C178 and C253 on the AQP4 intracellular domain.
• Other metal ions, including Cu^2+^, do not inhibit AQP4 in *in vitro* cell models.

## 1 Introduction

Aquaporin 4 (AQP4) is the most widely expressed aquaporin in the central nervous system (CNS) [1]. The expression of AQP4 in the CNS, at the blood brain barrier (BBB), makes it an important factor in many brain disorders, and a desirable target for pharmacological agents [2,3]. Aquaporin 4 has been implicated in neurological disorders including ischemia, stroke, and neuromyelitis optica [4]. For this reason identification of novel molecules which can modulate the activity of AQP4 is an ongoing topic of interest. The traditional methods for *in vitro* identification of compounds which can modulate the activity of an aquaporin include the oocyte swelling assay and reconstituted proteoliposome stopped flow assay [[5], [6], [7]]. In both of these assays, the aquaporin is inserted into a membrane, either via injection of mRNA into the oocyte leading to protein expression or physical insertion via proteoliposome reconstitution, and the speed at which water moves across the membrane is measured in response to an osmotic challenge [5,8]. To screen for novel compounds, the models are incubated with the molecule of interest prior to osmotic challenge, and the speed at which water crosses the membrane will either increase or decrease, allowing for the calculation of the osmotic water permeability coefficient, or P_f_ [9].

Several metal ions have been identified to inhibit AQP4 in *in vitro* models [10]. While previously AQP4 had been thought to be unable to be inhibited directly by metal ions, Yukutake et al. demonstrated that this was due to the binding sites for metal ions, in this case Hg^2+^, are located on the intracellular domain of AQP4, and therefore in the *Xenopus* oocyte model, these sites were not available to the Hg^2+^ ions [5]. This stresses the importance of using a proteoliposome model as well, where approximately 50% of the protein is inserted backwards, allowing for the intracellular domain of the protein to be exposed to the molecule of interest [11]. Using the proteoliposome model, Zn^2+^, Hg^2+^, and Cu^2+^ have all been shown to inhibit AQP4 [5,[12], [13], [14]], whereas in cell based models these molecules have no effect [15,16].

In order to screen for novel molecules we developed a new high throughput cell based method using Chinese Hamster Ovary (CHO) cell lines which stably express different forms of AQP4. We modified a previously published calcein self-quenching assay [17], and utilized a plate reader with an injector module to introduce the osmotic challenge and record an intensity time lapse over the course of 30 s per well. This method allows for up to 96 different conditions to be tested in similar conditions on the same plate, increasing the efficiency of compound screening. Using this method, we identified unique metal ion, Cu^1+^, which has the capacity to inhibit AQP4 in a cell based model, something which has not been described previously for metal ions. We also identify the likely residues which Cu^1+^ binds to, which are surprisingly on the intracellular domain of the protein, and discuss the implications of this discovery.

## 2 Methods

### 2.1 Protein synthesis and purification

AQP4 wild type and mutant form protein synthesis and purification were performed using the Bac-to-Bac baculovirus expression system (Thermo Fischer Scientific) as per the kit protocol. In brief, the cDNA of a HIS-tagged AQP4 form of interest was inserted into the pFastBac vector, and was transfected into MAX Efficiency™ DH10Bac™ Competent Cells (Thermo Fisher Scientific) for bacmid production and selection. Once a bacmid clone had been selected via PCR to check for insert, 1 μg of bacmid was transfected into a serum-free suspended culture of Sf9 cells at a concentration of 2–4x10^6^ cells/ml and p0 viral stock was harvested 96 h post transfection. 500 μl of p0 stock was added to 25 ml of serum-free suspended culture of Sf9 cells at 8 × 10^6^ cells/ml. 24–72 h later when cells showed clear signs of infection, cells were harvested and protein purification was performed using Ni-NTA Agarose (Qiagen) based column chromatography targeting the HIS-tag as previously described [18].

### 2.2 Proteoliposome reconstitution and water permeability assay

Proteoliposome reconstitution and water permeability assessment was performed as previously described [5]. Proteoliposomes were incubated with compounds of interest for 15 min prior to stopped-flow assay. Fluorescent intensity curves were analyzed in Matlab 2019b (Mathworks, Inc.). Statistical analysis was performed using RStudio version 1.1.463 (RStudio PBC). Data were analyzed using one-way ANOVA followed by the Tukey's test.

### 2.3 Generation of aquaporin expressing CHO cell lines

Wild type AQP4 cells were already available in house [19]. Creation of novel CHO cells lines was performed in the following manner. Naïve CHO cells were transfected on a 6 well plate with a linearized pIRES2-EGFP based plasmid containing the aquaporin sequence of interest and G418 resistance using Lipofectamine® 3000 (Thermo Fisher Scientific). 48 h post transfection cells were transferred into 10 cm dishes containing DMEM with G418 and allowed to grow to confluence. Cells were then diluted 1/10,000 and split into ten 10 cm dishes and left for 7–10 days to establish single cell derived colonies. Colony selection was performed based on cell morphology, strength of GFP signal, and purity of cell morphology and GFP expression within all cells in a colony. Selected colonies were transferred to a 96 well plate and grown to 10 cm dishes. Final selection of novel cell lines was performed using Western blot for protein expression, and water permeability and protein function using the CHO cell water permeability assay. Cell lines which had similar protein expression and protein function to the established wild type AQP4 CHO cells were chosen for each novel aquaporin cell line.

### 2.4 CHO cell water permeability assay

Cell water permeability was conducted via a modified calcein self‐quenching assay [17]. In brief, AQP4 expressing CHO cells were seeded at a density of 2.5 × 10^4^/well in a black sided, clear bottom 96‐well plate 48 h prior to conducting the permeability assay. On the day of the assay, cells were washed with 100 μl Hanks' balanced salt solution (HBSS), followed by incubation with 45 μl 20 μM Calcein AM (Thermo Fisher Scientific) in HBSS for 30 min at room temperature. Post calcein flooding, cells were washed once with 100 μl HBSS, and then incubated in 100 μl HBSS containing the molecule of interest. As CuCl is an unstable compound, fresh synthesis was performed 15 min prior to application to the cells via the reaction of Na_2_SO_3_ and CuCl_2_ to produce CuCl. The reaction was performed using a concentration of 10 mM CuCl_2_ and 50 mM Na_2_SO_3_, and dilutions were made of the result (10 mM CuCl and byproducts) in HBSS to test on the various CHO cell lines. The 96‐well plate was then loaded into a SpectraMax i3x (Molecular Devices, LLC.) for high osmotic concentration media injection and fluorescent intensity readings. 25 μl of 800mosm media was injected into each well (well volume 100 μl, osmolarity 300mosm) for an increase in osmotic concentration of 100mosm and intensity was read every 0.067s for 30s post injection at 23–25°C. Raw data were collected using softmax Pro 7.1 (Molecular Devices, LLC.) and imported into Matlab 2019b (Mathworks, Inc.) for analysis. P_f_ values were calculated in Matlab as previously described [5]. In brief, fluorescent intensity curves were imported into Matlab and a double exponential fit was applied to the raw data. The initial value of the derivative of the fitted curve was then used to calculate the P_f_ using the formula *P*_*f*_ *= (dV/dt)/(S*_0_*V_w_*Δc), where (dV/dt) refers to the change in volume over change in time, S_0_ refers to the initial surface area facing the external medium, V_w_ refers to the partial molal volume of water, and Δc refers to the change in osmotic concentration of the media. As the change in volume of a cell has a linear relationship to the change in intensity when using calciein self-quenching, the initial value of the derivative of the fluorescent intensity curve can be substituted in for the (dV/dt) value [17]. All other variables in the formula are constant depending on the method used. Statistical analysis was performed using RStudio version 1.1.463 (RStudio PBC). Data were analyzed using one-way ANOVA followed by the Tukey's test.

## 3 Results

### 3.1 Cu(I)Cl uniquely inhibits AQP4 in an *in vitro* CHO cell based model

**Fig. 1:**
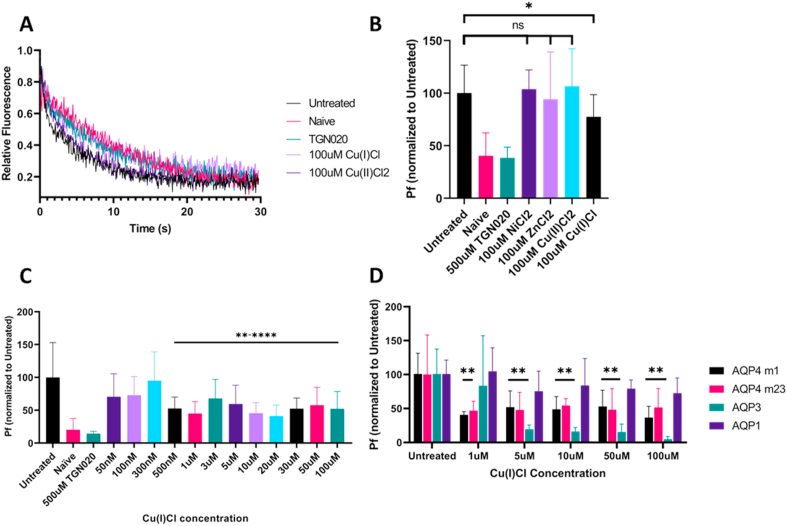
Cu(I)Cl inhibits AQP4 in a cell based model: (A) Representative calcein intensity curves of AQP4 expressing CHO cells exposed to TGN-020, Cu(II)Cl_2_, and Cu(I)Cl, as well as untreated and Naïve CHO cells. (B) P_f_ of AQP4 expressing CHO cells exposed to 100 μM NiCl_2,_ ZnCl, Cu(II)Cl_2_, and Cu(I)Cl (n = 8–24 per group). (C) Dilution series of Cu(I)Cl concentration ranging from 50nM to 100μM concentration (n = 8–24 per group). (D) Treatment of different cell lines expressing AQP4 m1, AQP4 m23, AQP3, and AQP1 with increasing concentrations of Cu(I)Cl. Asterisks indicate significant difference against untreated control (ANOVA followed by Tukey's test with 95% confidence, * = p < 0.05, ** = p < 0.01, **** = p < 0.0001).

The P_f_ of wild type M1 AQP4 was measured using a novel *in vitro* CHO cell model. Calcein stained cells were treated with different metal ions prior to exposure to an osmotic challenge. Fluorescent intensity curves were captured using a plate reader (Fig. 1A), and used to calculate the P_f_. While calcein is known to lose fluorescence under normal light over time, the entire assay from cell staining to results took place within 90 min and all cells were kept away from light for the duration of the experiment to avoid fluorescence loss. Post exposure to 100 μM concentrations of ZnCl_2_, and Cu(II)Cl_2_, known inhibitors of AQP4 in proteoliposome based models [13,14], and NiCl_2,_ a molecule we theorized may inhibit AQP4, no significant difference was found in P_f_ of the different treatment groups compared to the untreated control (Fig. 1B). However, after conversion of Cu(II)Cl_2_ to Cu(I)Cl via a reaction with 100 μM Na_2_SO_3_, 100 μM Cu(I)Cl was found to significantly lower the P_f_ of AQP4 in the cell based model (Fig. 1B, n = 24 decrease of 22.64% when compared to untreated control, p = 0.0031). 500 μM TGN-020, a known inhibitor of AQP4, was used as a positive inhibition control and decreased the P_f_ of AQP4 expressing cells by a mean of 61.63%, comparable with the Naïve CHO cell control (Fig. 1B). The side products of this reaction (2Cu(II)Cl_2_ + Na_2_SO_3_ + 2H_2_O = 2Cu(I)Cl + Na_2_SO_4_ + 2HCl), 100 μM HCl, 100 μM Na_2_SO_4_, and the complete reaction mixture (50 μM Na_2_SO_4_ + 50 μM Na_2_SO_3_ + 100 μM HCl) were tested in the same model and did not cause any significant decrease in P_f_, indicating that this inhibition is indeed caused by the Cu(I)Cl generated by the reaction (p-value = 0.012, Supplemental Fig. 1).

Cells were then treated with an extended dilution series of the Cu(I)Cl reaction, ranging from 100 μM to 50 nM, and a significant decrease of P_f_ was found in all groups above 500 nM concentration (Fig. 1C, n = 8–24 per group, inhibition ranged from 32.13% to 58.88% decrease compared to untreated). The Cu(I)Cl reaction mixture was tested on 4 different CHO cell lines, each expressing a different aquaporin. Cu(I)Cl was able to inhibit both the m1 and m23 forms of AQP4, as well as AQP3, while having no effect on AQP1, further suggesting that it is indeed the copper ion which is causing the inhibition, as Cu^2+^ has been shown in previous literature to inhibit AQP3 but have no effect on AQP1 [16].

### 3.2 Both Cu(I)Cl and Cu(II)Cl_2_ are able to inhibit AQP4 in a proteoliposome based model

**Fig. 2:**
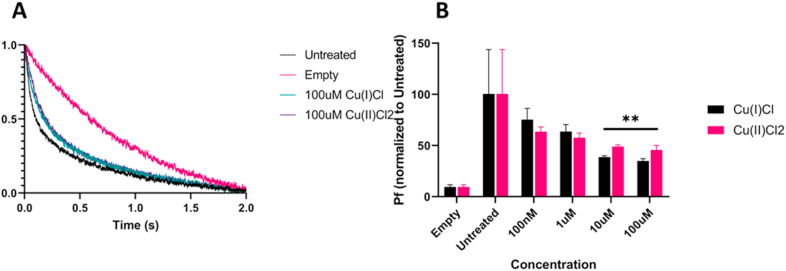
Proteoliposome based confirmation of Cu(I)Cl inhibitory activity: (A) Representative curves of proteoliposomes treated with 100 μM Cu(I)Cl and 100 μM Cu(II)Cl_2_. (B) P_f_ values of proteoliposomes treated with increasing concentrations of Cu(I)Cl and Cu(II)Cl_2_ (n = 10 per group). Asterisks indicate significant difference to the untreated controls (ANOVA followed by Tukey's test with 95% confidence, ** = p < 0.01).

To confirm the results of the CHO cell model, both forms of copper were tested in a proteoliposome based model. The advantage of this model is twofold; first it allows for determination of compound effect on the pure protein, and secondly it allows testing of compounds which bind to the intracellular domain, as approximately 50% of the protein will be inserted into the proteoliposome inverted. Carboxyfluorescein loaded proteoliposomes containing wild type AQP4 m1 were treated with either Cu(II)Cl_2_ or Cu(I)Cl for 15 min prior to analysis on a stopped flow apparatus (Fig. 2A).

Calculation of the P_f_ of each treatment group showed a dose-dependent inhibition of AQP4, with a slightly increased inhibition in the Cu(I)Cl treated proteoliposomes (Fig. 2B, n = 10). At the 100 μM concentration, Cu(I)Cl caused an average decrease of 65.13% in P_f_, and Cu(II)Cl_2_ caused an average decrease of 54.31% in P_f_ when compared with the untreated WT AQP4 proteoliposomes (Cu(I)Cl p < 0.0001, Cu(II)Cl_2_ p = 0.0007). Notably, in the proteoliposome model, both Cu(II)Cl_2_ and Cu(I)Cl were capable of inhibiting WT AQP4 m1.

### 3.3 Copper inhibits AQP4 by binding to C178 and C253

**Fig. 3:**
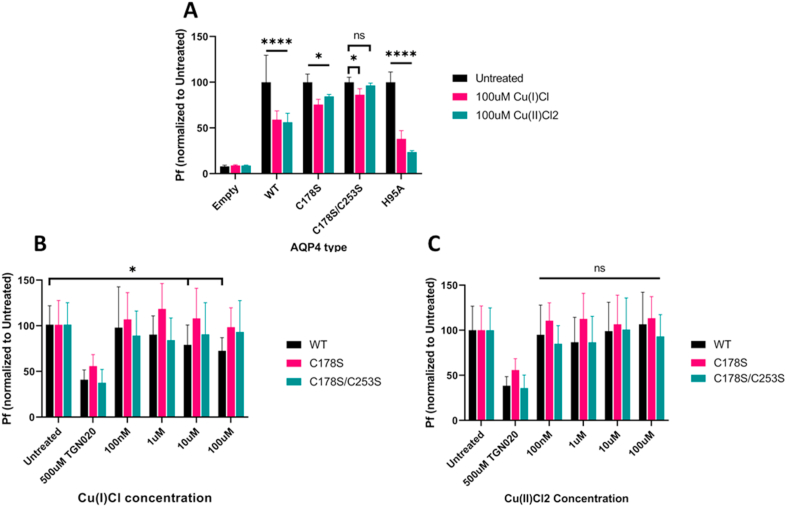
**Effect of copper on mutant AQP4 in proteoliposome and CHO cell *in vitro* models:** (A) P_f_ values of proteoliposomes containing the C178S, C178S/C253S, and H95A mutants of AQP4 treated with 100 μM Cu(I)Cl or 100 μM Cu(II)Cl_2_ (n = 10 per group). (B) P_f_ values of CHO cell lines expressing C178S and C178S/C253S mutant forms of AQP4 treated with 100 μM Cu(I)Cl (n = 24 per group). (C) P_f_ values of CHO cell lines expressing C178S and C178S/C253S mutant forms of AQP4 treated with 100 μM Cu(II)Cl_2_ (n = 24 per group). Asterisks indicate significant difference against untreated control groups (ANOVA followed by Tukey's test with 95% confidence, * = p < 0.05, ** = p < 0.01, **** = p < 0.0001).

In order to determine the mechanism of inhibition of copper on AQP4, mutant forms of AQP4 were generated. Three different mutants were tested in proteoliposomes: single mutant C178S, single mutant H95A, and double mutant C178S/C253S. While the H95A mutation did not have any effect on copper mediated inhibition of AQP4, the single C178S mutant showed a significant alleviation of the copper mediated inhibition, and the double mutant caused a near full recovery (Fig. 3A, n = 10 per group.) indicating that copper likely binds to both C178 and C253 in order to mediate the inhibitory effect. Intriguingly, even with the double mutant, Cu(I)Cl still caused a small but significant inhibitory effect (Figs. 3A and 13.7% inhibition, p = 0.007), which was not observed when Cu(II)Cl_2_ was used (p = 0.765).

With the proteoliposome results in mind, two CHO cells lines were generated which stably express the single mutant C178S and double mutant C178S/C253S AQP4, to confirm these results in the CHO cell model. Treatment of three cell lines; wild type AQP4 m1 expressing, C178S AQP4 expressing, and C178S/C253S AQP4 expressing with copper concentrations ranging from 100 nM to 100 μM showed a significant inhibition in WT AQP4 treated with Cu(I)Cl at concentrations of 10 μM and 100 μM, which was lost in both the single and double mutant AQP4 expressing cells (Fig. 3B, n = 24 per group). Worth noting, however, is that the double mutant AQP4 showed a non-significant inhibitory trend in all four groups, with pf decreases ranging from 84.24% to 93.14% (Fig. 3B). Consistent with the previously presented data, Cu(II)Cl_2_ did not show any significant inhibition in either WT or mutant AQP4 expressing cells (Fig. 3C, n = 24 per group). Due to its ability to bind to the extracellular domain of AQP4, TGN-020 could be used as a positive inhibition control for not only the WT but also the mutant AQP4 expressing cells (Fig. 3B and C), and proved effective in inhibiting water permeability through both mutant forms of AQP4.

## 4 Discussion

Several metal ions have been previously reported to inhibit AQP4, including Hg^2+^, Zn^2+^, and Cu^2+^ [12]. However, in each of these cases the inhibition was identified via the use of proteoliposomes, a model in which both the extracellular and intracellular domains of the protein are exposed to the compound of interest. In cell models, the majority of metal ions are unable to inhibit AQP4, as the binding sites for inhibition lie on the intracellular domain of the protein, and therefore are inaccessible to the compound of interest [2,5]. While some ions have been able to have an effect *in vivo* on AQP4, these all occurred due to cell signaling pathways and subsequent phosphorylation, rather than direct binding and inhibition itself [2]. For the first time, the results presented here show *in vitro* inhibition of AQP4 by a metal ion in a cell model without relying on pathways which are present *in vivo*. In the majority of publications investigating the effect of copper on aquaporins, Cu^2+^ is used [10,12,16], making this the first time Cu^1+^ has been investigated in the context of AQP4 activity modulation. There is an intriguing aspect to this data, however. In the CHO cell based model, Cu^1+^ was able to induce a decreased P_f_ whereas Cu^2+^, consistent with previous research, was not (Fig. 1). In contrast to this, both Cu^1+^ and Cu^2+^ are able to inhibit AQP4 in a proteoliposome model (Fig. 2). Interestingly, the potential for Cu^1+^ to restrict AQP4 activity seems significantly higher in the proteoliposome model when compared to the CHO cell model (Fig. 1, Fig. 2), which can be explained via ease of access to the protein binding sites. In the proteoliposome model, approximately 50% of the protein will be inserted in reverse orientation, exposing the internal binding sites to the Cu^1+^ in the media and allowing for easier direct binding, whereas in the case of the CHO cell model, Cu^1+^ must first cross the cell membrane to access the binding sites [5]. Furthermore, when the commonly known metal ion binding sites on AQP4, C178 and C253, were eliminated via point mutation, this inhibitory effect was, in the case of Cu^2+^, completely alleviated, and in the case of Cu^1+^, nearly completely alleviated (Fig. 3), strongly suggesting that these intracellular domains are the mechanism through which both copper isoforms are able to inhibit the permeability of AQP4. This suggests two possibilities. The first possibility is that Cu^1+^, with an increased binding potential, is able to bind to an extracellular domain of AQP4, which Cu^2+^ is not able to. Copper is known to inhibit AQP3 by binding to three residues on the extracellular domain of the protein. The first of two residues do not have a similar analogue on AQP4, however the third residue, AQP3 H241, lies in a very similar position to an AQP4 residue, H230, on the extracellular side of transmembrane helix 6 of AQP4 [16,20]. While the data in Fig. 3A, indicating that even a double mutant was not enough to fully alleviate inhibition of AQP4 via Cu^1+^ supports this possibility, in the cell model a double inhibition was statistically sufficient to completely rescue permeability post exposure to Cu^1+^, though Cu^1+^ still had a non-significant inhibitory effect (Fig. 3C), further bolstering the possibility of a third binding domain. The second possibility is that Cu^1+^ is able to cross the membrane and access the intracellular domain of AQP4 while Cu^2+^ does not have this same capability. This suggests the existence of a Cu^1+^ specific copper transporter. One such transporter is SLC31A1, an evolutionarily conserved Cu^1+^ transporter expressed on astrocytes in the immune system, and importantly reported to be expressed at the blood brain barrier [21,22]. While we were unable to conclusively determine whether SLC31A1 is the missing piece of the puzzle in the Cu^1+^ versus Cu^2+^ inhibition of AQP4 *in vitro* (Results not shown), it remains an interesting subject of study. It is worth noting that SLC31A1 is one of many proteins in the Cu^1+^ transporter family [23], and any one of the members expressed in mammalian cells may be responsible for active transport of Cu^1+^ in an *in vitro* cell model.

## 5 Conclusion

This report, for the first time, introduces a metal ion which is able to inhibit AQP4 by direct binding in a cell model *in vitro*. Cu^1+^, synthesized by reacting Cu(II)Cl_2_ with Na_2_SO_3_, is capable of inhibiting AQP4 in a cell model while Cu^2+^ was not. In contrast both Cu^1+^ and Cu^2+^ were able to decrease the P_f_ of AQP4 in a proteoliposome model, where both the intracellular and extracellular domains of the protein were exposed to the compound of interest. This inhibition was mediated by direct binding to C178 and C253 on the intracellular domain of the protein, indicating that Cu^1+^ is likely able to cross the cell membrane and access the intracellular domain of AQP4 *in vitro*, a phenomenon which has not been previously described for metal ion mediated inhibition of AQP4.

## Funding

This work was supported by grants from the 10.13039/501100001691Japan Society for the Promotion of Science Grant-in-Aid for Scientific Research (B) (18H02606); from Suntory Global Innovation Center Ltd. program “Water Channeling Life”; from 10.13039/501100001697Keio University Program for the Advancement of Research in Core Projects under Keio University's Longevity Initiative.

## Declaration of competing interest

Funding for the research from Suntory Global Innovation Center Ltd (M.Y).
