# Supplementary material for: Cu1+, but not Cu2+ is capable of inhibition of AQP4 permeability in an in vitro CHO cell based model
Source: Biochem Biophys Rep. 2021 Sep 14;28:101132. doi: 10.1016/j.bbrep.2021.101132 (PMC8446781; doi:10.1016/j.bbrep.2021.101132)
Supplement: Multimedia component 1 [file mmc1.docx]

**Supplementary Materials:**

**
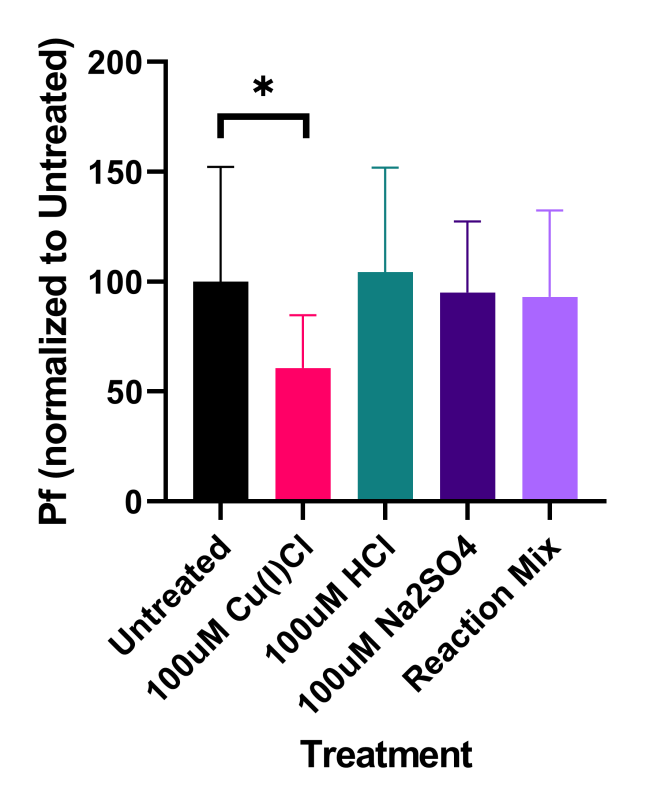
**

Supplementary Figure 1: Cu(I)Cl synthesis reaction byproduct testing. The byproducts of the Cu(I)Cl synthesis reaction were tested in the absence of Cu(I)Cl (n=8 per group). Reaction Mix refers to the final product without the presence of Cu(I)Cl. Asterisks indicate significant difference against untreated control groups (ANOVA followed by Tukey’s test with 95% confidence, *= p<0.05).
